# Effects of Wrist Posture and Fingertip Force on Median Nerve Blood Flow Velocity

**DOI:** 10.1155/2017/7156489

**Published:** 2017-02-13

**Authors:** Katherine E. Wilson, Jimmy Tat, Peter J. Keir

**Affiliations:** ^1^Occupational Biomechanics Laboratory, Department of Kinesiology, McMaster University, Hamilton, ON, Canada L8S 4K1; ^2^Department of Medicine, McGill University, Montreal, QC, Canada

## Abstract

*Purpose*. The purpose of this study was to assess nerve hypervascularization using high resolution ultrasonography to determine the effects of wrist posture and fingertip force on median nerve blood flow at the wrist in healthy participants and those experiencing carpal tunnel syndrome (CTS) symptoms.* Methods*. The median nerves of nine healthy participants and nine participants experiencing symptoms of CTS were evaluated using optimized ultrasonography in five wrist postures with and without a middle digit fingertip press (0, 6 N).* Results*. Both wrist posture and fingertip force had significant main effects on mean peak blood flow velocity. Blood flow velocity with a neutral wrist (2.87 cm/s) was significantly lower than flexed 30° (3.37 cm/s), flexed 15° (3.27 cm/s), and extended 30° (3.29 cm/s). Similarly, median nerve blood flow velocity was lower without force (2.81 cm/s) than with force (3.56 cm/s). A significant difference was not found between groups.* Discussion*. Vascular changes associated with CTS may be acutely induced by nonneutral wrist postures and fingertip force. This study represents an early evaluation of intraneural blood flow as a measure of nerve hypervascularization in response to occupational risk factors and advances our understanding of the vascular phenomena associated with peripheral nerve compression.

## 1. Introduction

Carpal tunnel syndrome (CTS) is the most prevalent work-related upper extremity disorder in Ontario, Canada [1]. A strong relationship exists between injury and work exposures, such as high force [2], high repetition [3], and awkward postures [4]. Characteristic signs and symptoms have been used to diagnose CTS, including pain, tingling, and numbness in the areas innervated by the median nerve [5]. The current reference standard for diagnosis is a nerve conduction study; however, this requires the nerve to have already suffered damage [6]. Sonographic techniques have proposed diagnosis criteria such as increased nerve cross-sectional area (CSA) and, more recently, alterations in the median nerve vasculature [7]. The increase in median nerve vascularization (hypervascularization) can be measured using high resolution ultrasonography of intraneural blood flow velocity. It is possible that vascular changes may occur within the median nerve prior to the characteristic signs of CTS, yet the underlying pathophysiology remains elusive. It has been theorized that compression leads to proximal nerve hypervascularization as a result of the distal ischemia caused by the entrapment [8]. Congestion in the epineural and endoneurial veins, nerve edema, and impairment of blood supply has been linked to the development of CTS [7]. This finding has led to the use of nerve hypervascularization as a possible new measure in the diagnosis of CTS.

Colour Doppler has been used to observe blood flow in the median nerve [7]. Due to technological advancement, pulse wave (PW) Doppler can now quantify nerve hypervascularization by measuring the intraneural blood flow velocity [9]. Strong correlations have been found between intraneural blood flow velocity and current diagnostic tests, such as nerve cross-sectional area [7]. A major advantage to this technique is that the hypervascularization component can be detected before the development of nerve swelling and edema, allowing much earlier detection of median nerve damage [7]. Applying this sonographic technique to examine vascular changes that occur throughout common work tasks is a novel concept that will be explored in this study.

Recent studies have quantified nerve hypervascularization in the neutral position for the purpose of diagnosis [6, 9]; the intent of the current study is to measure blood flow velocities under conditions that may place the nerve at risk of compression. The purpose of this study was to determine the effects of wrist posture and fingertip force on median nerve blood flow at the level of the wrist. The study also examined the vascular difference between healthy participants and those reporting carpal tunnel syndrome symptoms. Quantifying median nerve hypervascularization in response to common work tasks and activities of daily living may increase our understanding of the pathophysiology of CTS. This sonographic technique may prove to have value in the assessment of CTS risk in addition to its diagnostic value.

## 2. Materials and Methods

### 2.1. Participants

Nine participants experiencing the symptoms of carpal tunnel syndrome and nine healthy controls participated in this study (Table 1). There were fifteen females and three males with a mean age of 33 years (range 19–55). All participants used the right hand. All participants completed a questionnaire to screen for musculoskeletal disorders and health conditions that may influence the vascular or nervous system. Exclusion criteria for both groups included previous wrist surgery, radial malunion, Colles fracture, bifid median nerve, persistent median artery, degenerative joint disease, arthritis of the wrist/hand, gout, hemodialysis, sarcoidosis, amyloidosis, hypothyroidism, and diabetes mellitus. In addition, resting seated blood pressure was measured using automated oscillometry for all participants and any participants with abnormal blood pressure (above 140/90 mmHg) were excluded from the study. Each participant underwent a qualitative assessment using the Katz hand diagram [10], Phalen's Test [11], and Levine's CTS Questionnaire [12]. Only participants who were experiencing pain, tingling, or numbness of the hand were included in the CTS symptomatic group. The control group was required to have a negative Phalen's Test, a score of 0 on Levine's CTS Questionnaire, and a score of 0 on the Katz hand diagram. The study was approved by the Institutional Research Ethics Board.

### 2.2. Experimental Procedures

**Figure 1:**
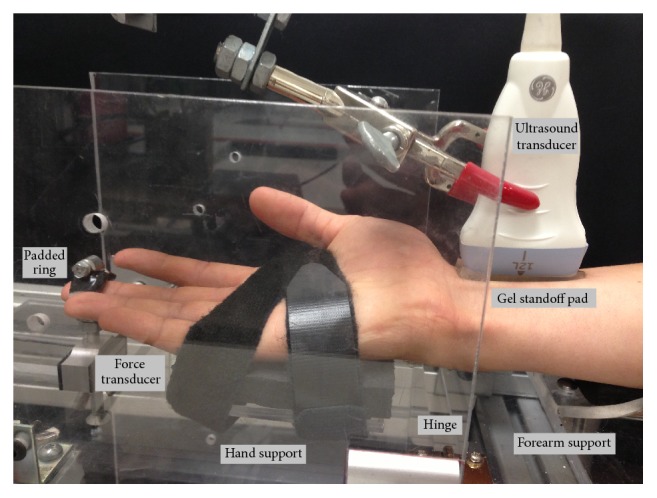
Experimental setup indicating hand and wrist posture, padded ring attached to force transducer, location of the hinge linking the forearm support and hand support, and the location of the ultrasound transducer (probe).

All participants performed the Katz hand diagram, Phalen's Test, and Levine's CTS Questionnaire at baseline. The Katz hand diagram was revisited throughout the study if there were changes in the participant's symptoms. Participants were seated with the right shoulder fully adducted and the right elbow positioned in a flexed posture. The right forearm was supinated and immobilized using a custom splint. The dorsal surface of the right hand was placed on a padded surface with a strap across the palm. To adjust the wrist posture, the forearm support was kept in a fixed position, while the hand support was attached to a hinge that secured the wrist into five postures (neutral, 15° flexion, 30° flexion, 15° extension, and 30° extension). All wrist postures were tested with and without the addition of a 6 N fingertip press based on previous studies of occupational tasks [13, 14]. The pulp of the middle finger was placed through an adjustable padded metal ring attached to a force transducer (MLP50, Transducer Techniques, Temecula, CA, USA) and the metal ring/force transducer moved the length of a slot to adjust for the participant finger length (Figure 1). Participants were instructed to press up with their fingertip. Visual feedback of their force was provided on a computer screen with a target line set at 6 N using a custom program (LabView 8.5, National Instruments, Texas, USA). Force was collected at 2000 Hz and reviewed after the trial to ensure the participants maintained the correct level of force (±15%) throughout the trial. Electrocardiography (ECG) was used to monitor heart rate throughout the study. Electrodes were placed on both clavicles and the left lower rib. The ECG was displayed on the sonographic system and aided in the analysis of the signal.

### 2.3. Sonographic Measurements

**Figure 2:**
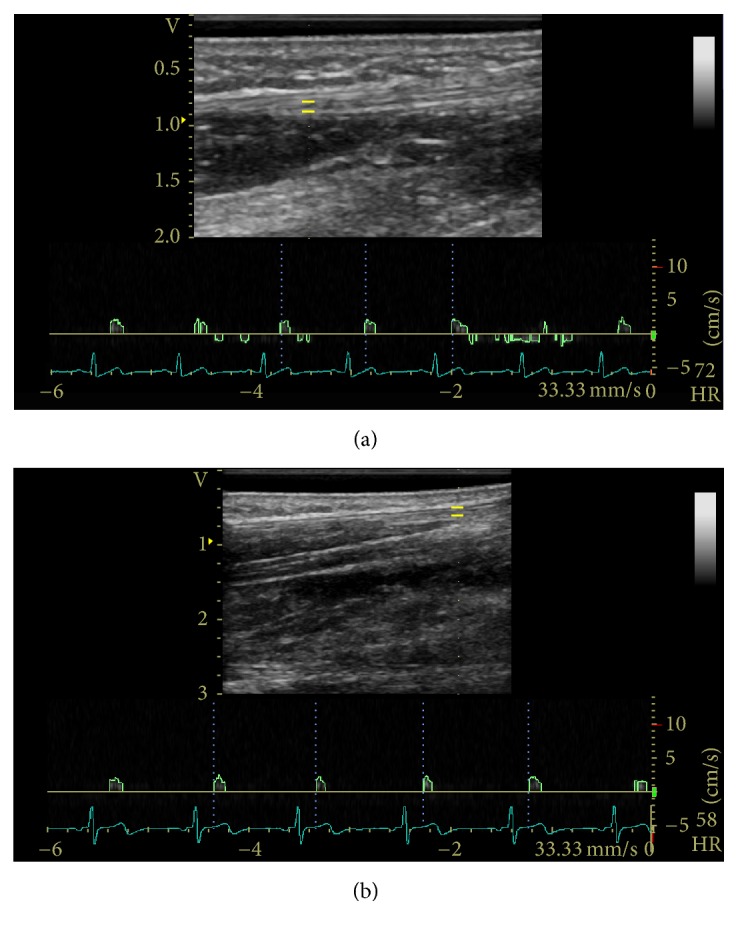
Sample PW Doppler examination in the (a) neutral position and (b) flexed 30° position with spectral tracing of the median nerve proximal to the wrist crease. The yellow bars indicate the gate or sample volume.

A Vivid Q BT10 sonographic system (General Electric Healthcare, Milwaukee, WI) equipped with a high frequency (12 MHz) linear array transducer was used to image the median nerve. Transverse images of the median nerve were taken by a trained examiner proximal to the carpal tunnel on the palmar surface at the level of the distal wrist crease and used to determine median nerve cross-sectional area (CSA). A neutral wrist posture was maintained and the fingers were kept in a semiflexed, relaxed position. Intraneural blood flow velocity was evaluated in the longitudinal plane with a custom gel standoff pad (thickness 2.5 mm; Aquaflex Gel Pad; Cone Instruments, Solon, OH) to provide a better nerve image at a greater depth. PW Doppler settings were standardised using a frequency of 12 MHz, pulse repetition frequency of 800 Hz, wall filter 0.3 cm/s, and a sample volume length of 0.98 mm. PW Doppler gain was optimized by increasing gain until noise appeared and then reduced just enough to suppress the noise. When the region with the highest area of blood was determined, the pulse wave gate was placed over the median nerve (Figure 2). Five seconds of steady state blood flow and consistent positive deflections was required. No steer angle or angle correction was used as the direction of flow was indeterminate. The initial peak systolic velocities were measured in the neutral position. Blood flow velocity was recorded after steady state was achieved in each of the wrist postures. Steady state was determined as a consistent signal over a five second interval. Participants rested between trials until blood flow returned its initial velocity. Trial order was block randomized by wrist posture. For all postures, participants performed the condition with no force followed by 6 N of force. The force was sustained for 3 minutes and the nerve blood flow was recorded throughout until blood flow had reached a steady state. All data was exported for further analyses (EchoPac postprocessing software, General Electric Healthcare, Milwaukee, WI). For a subset of 5 healthy participants, the full protocol was repeated two weeks later by the same examiner to test the reliability of the blood flow measurement.

### 2.4. Data Analysis

Phalen's Test was considered positive if participants reported pain, tingling, or numbness in the median nerve distribution over the one minute procedure [11]. The overall Levine's Symptom Severity and Functional Status score was calculated as the mean of the scores for each section [12]. Katz hand diagrams were scored independently by two researchers masked to the participant's performance in the study [10, 15]. The nerve CSA was measured from the transverse images using electronic calipers to outline the internal rim and rounded to the nearest 0.01 cm^2^. The mean peak blood flow velocity of all participants for each posture and force level was determined as the mean peak velocity (cm/s) of a consistent 5 s steady state.

### 2.5. Statistical Analysis

A repeated measures mixed design ANOVA was performed on the mean peak blood flow velocities. Post hoc comparisons were conducted with Tukey's HSD tests. Pearson product-moment correlations were performed on all qualitative assessments, nerve CSA, and the mean peak blood flow velocities. Reliability of mean peak blood flow velocities was tested in five healthy participants using intraclass correlations (ICC) and Bland-Altman plots. The mean peak velocity of each testing condition was compared between day 1 and day 2. All statistical analyses were performed using SPSS Statistics 17.0 with significance level set at *p* < 0.05.

## 3. Results

### 3.1. Effect of Wrist Posture and Fingertip Force

**Figure 3:**
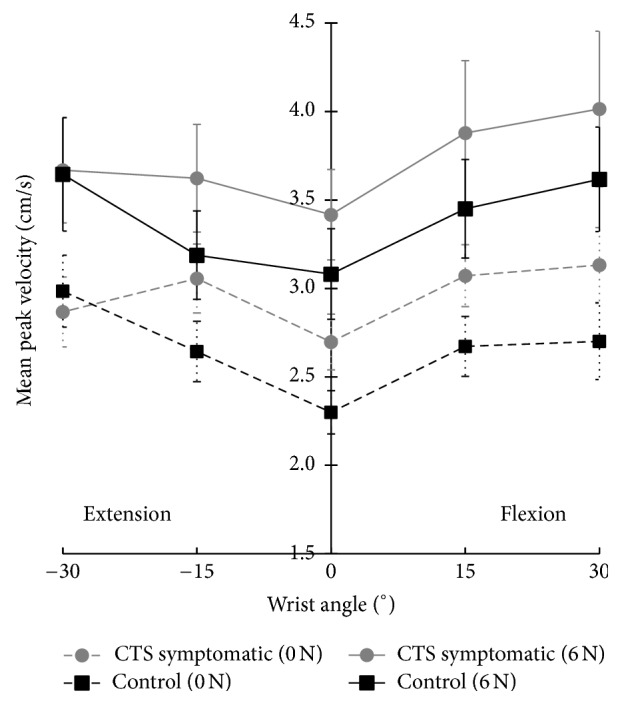
Mean peak velocity (cm/s) with standard error of the intraneural blood flow of the median nerve at the proximal level of the carpal tunnel in controls and CTS symptomatics with and without a 6 N fingertip force in five wrist postures.

**Figure 4:**
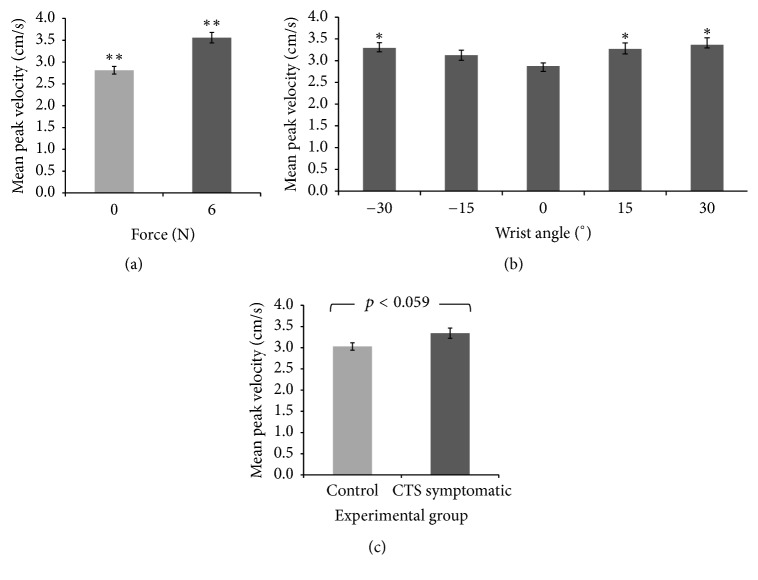
Mean peak intraneural blood flow velocity (cm/s) with standard error to illustrate (a) a force main effect (^*∗∗*^significantly different, *p* < 0.0005), (b) a wrist posture main effect (^*∗*^significantly greater than 0°, *p* < 0.02), and (c) a general trend in the experimental group (*p* = 0.059).

Two main effects (wrist posture and fingertip force) and a trend (experimental group) are shown in Figure 3; no interaction effects were found. There was a significant main effect of force on blood flow velocity (*F*_1,16_ = 28.039, *p* < 0.0005). The mean peak blood flow velocity was greater with force (3.56 cm/s) than without force (2.81 cm/s) (Figure 4(a)). This was consistent across all wrist postures in both experimental groups. A significant main effect of wrist posture was found (*F*_4,64_ = 3.163, *p* < 0.02; Figure 4(b)). Deviated wrist postures produced greater blood flow velocities than the neutral wrist posture. Wrist postures of flexion 30° (3.37 cm/s), flexion 15° (3.27 cm/s), and extension 30° (3.29 cm/s) had significantly higher blood flow velocities than the neutral (2.87 cm/s) wrist posture (all *p* < 0.002). The blood flow velocity in extension 15° (3.13 cm/s) was also higher than neutral but did not reach statistical significance (*p* = 0.10). Additionally, there was a significant quadratic relationship between wrist posture and intraneural blood flow velocity (*p* < 0.0005). In a separate analysis, the CSA of the median nerve at the distal wrist crease in the neutral position significantly correlated with the peak blood flow velocity at neutral with no force (*r* = 0.495, *p* = 0.04).

### 3.2. Vascular Difference between Healthy and CTS Symptomatic Groups

The mean peak blood flow velocity of CTS symptomatic group (3.34 cm/s) was slightly higher than the healthy controls (3.03 cm/s) but the difference was not statistically significant (*F*_1,16_ = 4.121, *p* = 0.06) (Figure 4(c)). Descriptive statistics showed that the baseline (neutral, no force) velocity in the CTS symptomatic group was generally higher than the control group; however, this was not consistent for all participants. In a separate analysis, the CTS symptomatic group, although not clinically diagnosed, showed a significant correlation with four commonly used diagnostic tools: a positive Phalen's Test (*r* = 0.535, *p* < 0.02), a higher Katz hand diagram score (*r* = 0.620, *p* < 0.006), a higher Levine's Symptom Severity score (*r* = 0.925, *p* < 0.0005), and a higher Levine's Functional Status score (*r* = 0.730, *p* < 0.001). In addition, the Katz hand diagram was used to document symptoms after each trial and the results showed that the CTS symptomatic group was more prone to symptoms than the control group. Pain and tingling were the most common symptoms.

### 3.3. Reliability Analysis

**Figure 5:**
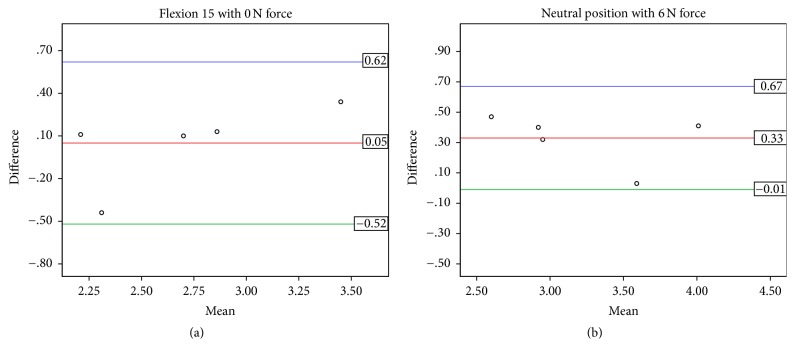
Distribution plots from Bland-Altman analyses for between-days repeatability (*N* = 5) of mean peak blood flow velocity (cm/s) in (a) 15° flexion without force and (b) neutral wrist with 6 N force. The *x*-axis represents the between-days mean measurement. The *y*-axis represents the between-days difference between measurements. The mean line represents the mean difference between days with the upper and lower lines representing the limits of agreement (1.96 SD).

In a subset of five participants from the control group, the mean peak velocity (cm/s) between day 1 and day 2 demonstrated reasonable agreement (Table 2). Between-days repeatability was high for neutral with force (ICC 0.818, 95% confidence interval (CI): 0.149, 0.979) and 15° of flexion without force (ICC 0.868, 95% CI 0.313, 0.985). The Bland-Altman plots for the two conditions with the high ICCs, neutral wrist with 6 N force and 15° of flexion without force, are shown in Figure 5. Repeatability was moderate to fair for the remaining conditions (Table 2).

## 4. Discussion

The pathophysiology of nerve hypervascularization remains unclear but this study sheds insight on the role of posture and force on the vascular system of the median nerve. This study examined the effect of two common work tasks on the median nerve blood flow as it enters the carpal tunnel. Blood flow was successfully quantified in five wrist postures with two force levels. Previously, intraneural blood flow velocity had only been reported in the neutral posture. A positive quadratic relationship was found between median nerve blood flow velocity and deviated wrist postures. We confirmed our hypothesis that both flexed and extended postures would increase blood flow above neutral. Compared to neutral, increased blood flow was found with 30° extension (+0.42 cm/s) and 15° flexion (+0.39 cm/s) with the greatest increase at 30° flexion (+0.49 cm/s). Though not statistically significant (*p* = 0.10), 15° extension also showed a higher velocity (+0.25 cm/s) than neutral. Blood flow velocity had a positive relationship with force. We confirmed our hypothesis that fingertip force would increase blood flow in all wrist postures. The 6 N force increased the velocity by 0.75 cm/s, significantly higher than without force. Moreover, a proximal increase in vascularity in the neutral position has been shown to have a positive correlation with nerve CSA due to nerve enlargement and edema as a result of CTS [10, 16]. Our study corroborated a positive correlation between nerve CSA and peak blood flow velocity at baseline.

Based on previous findings, blood flow was expected to be higher in the CTS symptomatic group [7]. Our results did not show a statistically significant finding; however it showed higher mean velocities with those experiencing CTS symptoms (+0.31 cm/s) than those without symptoms. Participants were categorized using the three qualitative assessments (Katz hand diagram, Phalen's Test, and Levine's CTS Questionnaire); however, no strong correlations were found with baseline (neutral, no force) velocity data. However, this does not discount the potential of intraneural blood flow velocity as a diagnostic aid. Joy et al. found significant pathological differences in blood flow with severe CTS patients [9] and Mallouhi et al. reported more reliable evaluation of velocity in nerve conduction diagnosed patients [7]. It is noteworthy that, despite this study's CTS symptomatic group being self-diagnosed and exhibiting mild signs of CTS, we found higher velocity in the CTS symptomatic group (*p* < 0.06) which also correlated well with four common clinical tests (*p* < 0.05). Additionally, we tested the reliability of the technique by repeating the protocol after two weeks in five controls. Reasonable agreement was found using Bland-Altman plots and strong ICCs in some conditions (Table 2 and Figure 5), suggesting promising use of this method in future research. However, a fair ICC for some testing conditions indicates that more investigation of this technique is needed. It is yet to be determined if the unknown variability may be due to the methodology of the protocol or variability within individual blood flow velocities. Support for the methodology is seen in the mean changes due to posture (Figure 3) that are similar in pattern within each condition (0.5–0.7 cm/s) with larger step increases between conditions (0.4–1.2 cm/s). These changes are larger than the 2 standard deviation lines in the Bland-Altman plots (Figure 5) suggesting strength in the techniques used despite using healthy and symptomatic participants in which these responses may be less pronounced. With more research, these findings suggest that hypervascularization may have potential to provide an objective measure of injury to the nerve prior to demyelination occurring. Further research with clinically diagnosed CTS patients and a larger scale reliability study may help clarify this relationship.

The 10% difference in velocity between the control and CTS symptomatic groups suggests a compounded, potentially pathological, increase in blood flow found with deviated wrist posture and fingertip force. The acute stages of nerve compression are thought to be primarily vascular with damage to the blood-nerve barrier endoneurial capillaries leading to edema and increase in fluid pressure [17]. Rydevik and Lundborg (1977) found evidence of epineurial edema within 2 hours of 50 mmHg pressure, demonstrating that high CTP over a short period of time causes changes within the carpal tunnel [18]. It is well documented that deviated wrist postures and fingertip force can dramatically change the carpal tunnel fluid pressure [10, 13, 19, 20]. Potentially, hypervascularization (increased flow) combined with venous congestion (lack of fluid release) may cause the increase in CTP found with deviated wrist posture and/or fingertip force [21]. Likening carpal tunnel dynamics to a closed compartment supports the construct that an immediate increase in blood flow is necessary to cause the acute edema seen in CTS patients. Our results suggest that these factors may acutely induce vascular changes associated with CTS. The trend of higher velocities in the CTS symptomatic group and transient symptoms triggered in some control participants suggests that a pathological response and pathological vasodilation are occurring. Further, the fingertip force triggered symptoms in the CTS symptomatic group, supporting similar findings by Rempel et al. [21]. With further research, this technique may have potential in early diagnosis of CTS before the distal nerve becomes ischemic and severely damaged and has applications to elucidate the role of occupational risk factors in the development of chronic median nerve compression. Furthermore, the risk of developing CTS can be evaluated with some of the results of this study. The current data indicate that the stress on the median nerve during deviated wrist postures and fingertip force may affect even the healthy intraneural vascular system. With greater duration, deviated wrist postures may prove to be detrimental to the nerve regardless of experimental group. Furthermore, a relatively small 6 N force was used in this study, yet it still elicited a detectable change in blood flow velocity in both groups, suggesting that a low force threshold held for several minutes may be a risk factor for median nerve compression. It is thought that higher forces may prove more detrimental. Interestingly, we found no interaction between wrist posture and fingertip force supporting a previous pressure study which found these factors to act independently [21].

A limitation of this study was using PW Doppler to find intraneural blood flow velocity. Maintaining an image of the same median nerve segment throughout each posture was difficult due to slight longitudinal shifts of the proximal forearm with posture, requiring the transducer to be repositioned. Additionally, angle correction was not used as the direction of blood flow was indeterminate. However, no correction was used for all conditions allowing within-subject comparisons. Our CTS symptomatic group was low severity and may limit interpretation of the findings. A higher frequency transducer may also allow the low velocities of less severe participants to be more easily detected and better depict the vascular structures within the nerve.

This study demonstrates that measuring intraneural blood flow velocity using ultrasonography is a feasible technique. The results warrant further exploration of the role of intraneural vascular pressure in CTS and assessing PW Doppler as a potential tool to quantify intraneural blood flow velocity. The reliability of the technique shows a need for more research and a large scale reliability test should be explored. Median nerve vascularity will need to be investigated further in the assessment of individuals with mild and severe CTS. The evaluation of these common work tasks in clinically diagnosed CTS patients will be a valuable addition to this research.

## Figures and Tables

**Table 1:** Characteristics of study participants including qualitative (clinical) CTS tests.

Variable	Control (*N* = 9)	CTS symptomatic (*N* = 9)
Age^*∗*^	29 (10)	36 (15)
Gender	1 M, 8 F	2 M, 7 F
CSA at inlet (cm^2^)^*∗*^	0.10 (0.04)	0.09 (0.02)
Right hand dominance	9	9
Levine's Symptom Severity score^*∗*^	0	1.75 (0.54)
Levine's Functional Status score^*∗*^	0	1.32 (0.93)
Phalen's Test (+)	0	4
Katz hand diagram (>0)	0	6

^*∗*^Mean with standard deviation.

**Table 2:** Results of the intraclass correlation and Bland-Altman analyses.

		Intraclass correlation			Bland-Altman			
		Intraclass correlation coefficient	95% confidence interval		Mean difference	SD of the differences	95% confidence interval	
			Lower bound	Upper bound			Lower bound	Upper bound
0 N	Neutral	.336	−.572	.899	−0.01	0.37	−0.74	0.72
	Flex 15°	.868	.313	.985	0.05	0.29	−0.52	0.62
	Flex 30°	−.501	−.914	.514	−0.17	0.81	−1.77	1.42
	Ext 15°	−.109	−.804	.765	0.04	0.72	−1.37	1.44
	Ext 30°	.379	−.538	.908	−0.13	0.47	−1.05	0.79

6 N	Neutral	.818	.149	.979	0.33	0.17	−0.01	0.67
	Flex 15°	.077	−.727	.832	−0.11	0.76	−1.59	1.38
	Flex 30°	−.115	−.806	.763	0.02	1.24	−2.41	2.45
	Ext 15°	−.080	−.793	.777	0.32	0.64	−0.95	1.58
	Ext 30°	.342	−.568	.900	−0.34	0.75	−1.81	1.13
